# A rare case of diabetic ketoacidosis presenting with severe hypertriglyceridemia requiring plasmapheresis in an adult with type-2 diabetes mellitus: Case report

**DOI:** 10.1097/MD.0000000000026237

**Published:** 2021-06-11

**Authors:** Pooja Roy, Paige Koetter, Jessica Cunningham, Saketram Komanduri, John Cinicola

**Affiliations:** a Internal Medicine Residency Program, UPMC Pinnacle Harrisburg Hospital; b Penn State College of Medicine, Hershey, PA.

**Keywords:** case report, diabetic ketoacidosis, plasmapheresis, severe hypertriglyceridemia

## Abstract

**Introduction:**

Severe hypertriglyceridemia (HTG) is a rare complication of insulin resistance. Its presentation with diabetic ketoacidosis (DKA) has been reported in a few cases, where most patients have type-1 diabetes mellitus (DM). Our case represents a unique presentation of DKA associated with severe HTG above 10,000 mg/dL in an adult with type-2 DM.

**Patient concerns and diagnosis:**

Case Report: A 51-year-old man with no prior illnesses presented to the emergency department with abdominal pain and nausea. He was found to have DKA with a blood glucose level of 337 mg/dL, pH of 7.17, beta-hydroxybutyrate of 7.93 mmol/L, and anion gap of 20 mmol/L. His triglyceride levels were >10,000 mg/dL. His serum was found to be lipemic. Computerized tomography scan of the abdomen demonstrated mild acute pancreatitis. Negative GAD65 antibodies supported the diagnosis of type-2 DM.

**Interventions and outcomes:**

Endocrinology was consulted and one cycle of albumin-bound plasmapheresis was administered. This therapy significantly improved his HTG. DKA gradually resolved with insulin therapy as well. He was discharged home with endocrinology follow-up.

**Conclusion:**

This unique case highlights an uncommon but critical consequence of uncontrolled DM. It brings forth the possibility of severe HTG presenting as a complication of uncontrolled type-2 DM. Severe HTG commonly presents with acute pancreatitis, which can be debilitating if not managed promptly. Most patients with this presentation are managed with insulin infusion. The use of plasmapheresis for management of severe HTG has not been well studied. Our case supports the use of plasmapheresis as an effective and rapid treatment for severe HTG.

## 1 Introduction

Hypertriglyceridemia (HTG) is defined as triglyceride levels of >150 mg/dL (>1.7 mmol/L) and further classified into moderate and severe HTG with defining levels of 150 to 885 mg/dL and above 885 mg/dL, respectively.^[1]^ The etiology of HTG is multifactorial, and includes insulin resistance disorders, certain medications, that is, estrogens and bile sequestrants, and renal disease. However, severe HTG is mostly attributed to genetic causes such as familial chylomicronemia or type V hyperlipoproteinemia.^[1]^ Clinical manifestations of HTG vary based on its severity and etiology. Xanthomas and xanthelasmas are commonly seen in patients with hereditary disorders. Pancreatitis is another common presentation, with the risk of development between 10% and 20% in those with triglyceride levels of >2000 mg/dL.^[1]^ In rare cases, patients with HTG-induced pancreatitis present with diabetic ketoacidosis (DKA). In these cases, in which DKA induced HTG, triglyceride levels usually do not exceed 1500 mg/dL.^[2,3]^ There are 2 reported cases where triglyceride levels exceed 10,000 mg/dL.^[4,5]^ In both of these known cases, the patients were young and had a history of type-1 diabetes mellitus (DM).^[4,5]^ We present a unique case of DKA-induced severe HTG in a middle-aged adult without type-1 DM.

## 2 Case

The patient is a 51-year-old Hispanic man with no past medical history who presented to the emergency department with abdominal pain and nausea. His symptoms started 2 weeks prior with a burning sensation during urination, associated with polydipsia and polyuria. Three days later, he developed abdominal pain in the periumbilical region. The pain was nonradiating and continued to worsen until he was unable to eat. This was associated with nausea and constipation. He denied fevers or chills. He had no history of abdominal surgery or family history of diabetes or lipid disorders. His body mass index was 26.34 kg/m^2^ and he consumed 6 to 12 beers on the weekend. His examination findings were only significant for left upper quadrant abdominal tenderness. The patient was found to have severe HTG, with levels reported in Table 1. Low-density lipoprotein (LDL) and very low-density lipoprotein (VLDL) levels could not be calculated due to elevated triglycerides. In addition, the patient was hyperglycemic, with a blood glucose of 337 mg/dL and a glycated hemoglobin of 14.9%. An elevated anion gap metabolic acidosis was present with increased serum ketones, suggestive of DKA (Table 1). GAD-65 antibody was negative (<5 IU/mL), making type-1 diabetes less likely. Patient's islet cell antibody screen was not performed as the specimen was hyperlipemic. Urinalysis revealed a urine glucose of >1000 mg/dL and mild ketonuria (40 mg/dL). Lactic acid was mildly elevated at 2.6 U/L. Though lipase level was normal (43 U/L), computerized tomography scan of the abdomen demonstrated findings suggestive of mild acute pancreatitis. To note, the patient's serum was found to be milky in appearance. The patient was started on insulin and potassium chloride infusions and was transferred to the intensive care unit. After consulting endocrinology, one session of plasmapheresis was performed, which improved the patient's triglycerides gradually to 323 mg/dL. Fenofibrate was administered. The patient's blood glucose improved and subsequently he was transitioned to subcutaneous insulin. Triglyceride and cholesterol levels improved, as reported in Table 1. In addition, the apolipoprotein profile suggested an increased risk of atherosclerosis (Table 1). The patient was stable to be discharged with follow up with his primary care physician and endocrinologist.

**Table 1:** Laboratory results during the course of patient's admission.

	8/24/20	8/25/20	8/26/20 (plasmapheresis administered)	8/27/20	8/28/20	Ref. range
Triglyceride (mg/dL)	>10,000	3,823	913	423	323	0–150
Cholesterol (mg/dL)	1040			306		0–200
HDL (md/dL)	24			14		23–92
pH		7.17				7.35–7.45
Serum CO_2_ (mm Hg)		22				35–45
Serum bicarbonate (mmol/L)	14	6	14.3	20.5	27.3	19–33
Anion gap (mmol/L)	22	20	12.7	5.5	3	4–12
Beta-hydroxybutyrate (mmol/L)		7.93				0–0.3
Apolipoprotein A1					48	>115
Apolipoprotein B					113	<90
A1/B100 ratio					2.35	<0.77

## 3 Discussion

According to the National Health and Nutrition Examination Survey, only <1% of American adults not treated with statins had triglyceride levels >1000 mg/dL.^[6]^ There are also ethnic differences in triglyceride levels, with non-Hispanic blacks having lower triglyceride levels than non-Hispanic whites and Mexican Americans.^[7]^ Diabetic ketoacidosis, however, is a common complication amongst type-1 diabetics, with cases amongst type-2 diabetics rising.^[8]^ DKA is associated with an increase in triglyceride levels, observed in approximately 30% to 50% of cases.^[9]^ Most cases have triglyceride levels of <1000 mg/dL.^[2,3]^ In reported cases, patients with triglyceride levels higher than 10,000 mg/dL were younger than 21-years-old and had a history of type-1 DM (Table 2).^[4,5]^

**Table 2:** Literature review of cases of severe hypertriglyceridemia.

Title of article	Age	DM status	Triglyceride (mg/dL)
Severe hypertriglyceridemia: a rare and harmful complication in diabetic ketoacidosis, treated successfully with plasmapheresis (7)	14	Type 1	14,820
Severe hypertriglyceridemia in Diabetic ketoacidosis accompanied by acute pancreatitis: case report (8)	20	Type 1	15,240
Hypertriglyceridemia-induced acute pancreatitis with diabetic ketoacidosis: a rare presentation of type 1 diabetes mellitus (4)	23	Type 1	1100
Triad of diabetic ketoacidosis, hypertriglyceridemia, and acute pancreatitis: severity of acute pancreatitis may correlate with the level of hypertriglyceridemia (9)	50	Type 2	1226

The pathophysiology behind DKA-induced HTG is based on insulin resistance or deficiency. When the body lacks insulin or is resistant to insulin, lipolysis occurs, leading to the release of free fatty acids.^[4]^ The increased uptake of free fatty acids into the liver results in a greater production of VLDL, which is eventually converted to triglycerides. Insulin deficiency impedes the activity of lipoprotein lipase, thus interfering with triglyceride metabolism and VLDL catabolism.^[10]^

Severe HTG increases a patient's risk of acute pancreatitis. This disease can be devastating, with complications such as septic shock and multiorgan failure. Chronically elevated levels of triglyceride are also associated with a higher risk of atherosclerotic cardiovascular disease.^[1]^ Hence it is vital that severe HTG is promptly managed.

The management of HTG begins with lifestyle modifications. Recommendations include weight loss, dietary changes and avoidance of alcohol. Those with moderate triglyceride levels, between 150 and 885 mg/dL, do not have a significant risk of pancreatitis. Thus, these patients can be started on statins to reduce their risk of atherosclerotic cardiovascular disease.^[1]^ If triglyceride levels are persistently high, pharmacological intervention is warranted, beginning with fibrates, which are shown to lower triglyceride levels up to 70%.^[11]^ Fenofibrate is generally preferred over gemfibrozil due to the increased risk of muscle toxicity when used together with statins.^[1]^ In addition, according to the REDUCE-IT trial, omega-3 agents can further reduce triglyceride levels by 15% to 35%.^[12]^ Rarely, plasmapheresis is required to treat HTG acutely. The American Society of Apheresis’ 2016 guidelines recommend the use of plasmapheresis in HTG-induced pancreatitis (Grade 2C).^[13]^ The mechanism is such that during filtration, the passage of high molecular weight molecules such as triglycerides is prevented.^[4]^ The evidence for this therapy is currently not substantial.

There were limitations to our patient evaluation and management. No genetic studies or additional antibody tests, such as islet antigen 2 or insulin autoantibody measurements were performed, which could have provided diagnostic evidence of type-1 DM and lipid disorders. Additionally, we could not exclude HTG triggering DKA. In vitro studies by Garg et al,^[10]^ have shown that “high concentrations of triglyceride-rich CLDL particles may impair insulin action by inhibiting insulin binding to its receptor.” This could imply that insulin-resistance can occur secondary to hyperlipidemia.

## 4 Conclusion

In summary, our case represents a rare occurrence of severe HTG with DKA in an adult patient without a history of type-1 DM. This case encourages routine testing lipid panels in those with uncontrolled DM. Though evidence may be lacking, plasmapheresis was effective for this patient. Hence, this case supports the expanded use of plasmapheresis in patients with similar presentations of severe HTG.

## Acknowledgments

I would like to thank my mentors for helping me finalize this report and to Paige, the medical student who helped me with the literature review.

## Author contributions

**Conceptualization:** Pooja Roy.

**Data curation:** Pooja Roy.

**Investigation:** Paige Koetter.

**Resources:** Pooja Roy.

**Supervision:** John Cinicola.

**Validation:** Pooja Roy.

**Visualization:** Pooja Roy.

**Writing – original draft:** Pooja Roy.

**Writing – review & editing:** Pooja Roy, Jessica Cunningham, Saketram Komanduri.
